# Elucidating type 2 diabetes mellitus risk factor by promoting lipid metabolism with gymnemagenin: An *in vitro* and *in silico* approach

**DOI:** 10.3389/fphar.2022.1074342

**Published:** 2022-12-13

**Authors:** Anusree DasNandy, Vishal S. Patil, Harsha V. Hegde, Darasaguppe R. Harish, Subarna Roy

**Affiliations:** Indian Council of Medical Research-National Institute of Traditional Medicine, Belagavi, Karnataka, India

**Keywords:** gymnemagenin, lipid metabolism, obesity, *in vitro* study, *in silico* pharmacology, type 2 diabetes mellitus

## Abstract

**Introduction:** Adipose tissue functions as a key endocrine organ which releases multiple bioactive substances and regulate obesity-linked complications. Dysregulation of adipocyte differentiation, triglyceride metabolism, adipokines production and lipid transport contributes to impaired lipid metabolism resulting in obesity, insulin resistance and type 2 diabetes. *Gymnema sylvestre* plant is frequently used in Ayurveda for treatment of diabetes and obesity. Gymnemagenin is a major bioactive compound of *Gymnema sylvestre*. The present study was undertaken to elucidate the role of gymnemagenin in lipid metabolism by *in vitro* and computational approaches.

**Methods:** A panel of twelve genes viz., *Fasn, Lipe, Lpl, Pparg, Plin2, Cidea, Scd1, Adipoq, Lep, Ccl2, Fabp4, and Slc2a4,* essential in lipid metabolism were selected and gene expression pattern and triglyceride content were checked in adipocytes (3T3L1 cells) with/without treatment of gymnemagenin by Real time PCR and colorimetric estimation, respectively. Mode of action of gymnemagenin on Pparg and Fabp4 was accomplished by computational studies. Gene set enrichment and network pharmacology were performed by STRING and Cytoscape. Molecular docking was performed by AutoDock vina by POAP pipeline. Molecular dynamics, MM-PBSA were done by Gromacs tool.

**Results:** *In vitro* study showed that gymnemagenin improved triglyceride metabolism by up regulating the expression of lipase genes viz., *Lipe and Lpl* which hydrolyse triglyceride. Gymnemagenin also up regulated the expression of anti-inflammatory gene *Adipoq*. Importantly, gymnemagenin treatment up regulated the expression of *Pparg* gene and the downstream target genes (*Plin2, Cidea, and Scd1*) which are associated with adipogenesis. However, gymnemagenin has no effect on expression of *Fabp4*, codes for a lipid transport protein. *In silico* study revealed that gymnemagenin targeted 12 genes were modulating 6 molecular pathways involved in diabetes and obesity. Molecular docking and dynamics revealed that gymnemagenin stably bind to active site residue of Pparg and failed to bind to Fabp4 active site compared to its standard molecules throughout 100 ns MD production run. Gymnemagenin scored binding free energy of −177.94 and −25.406 kJ/mol with Pparg and Fabp4, respectively.

**Conclusion:** Gymnemagenin improved lipid metabolism by increasing triglyceride hydrolysis (lipolysis), up regulating the crucial gene of adipogenesis and increasing the expression of anti-inflammatory adipokine proving its therapeutic importance as anti-obesity and anti-diabetic phytocompound.

## 1 Introduction

Type 2 diabetes (T2D) is a prevalent metabolic disorder. More than 95% of people having diabetes are diagnosed with T2D. T2D is commonly asymptomatic and frequently recognized by the manifestation of excess body weight and elevation of random blood glucose. It is often diagnosed several years after the onset, when many other complications have already arisen (Cole and Florez, 2020). T2D starts with the onset of insulin resistance, which is a cumulative health consequence of obesity, dysfunctional adipose tissue, chronic inflammation, and decrease in pancreatic β-cell mass and consecutive failure in the production of insulin. In addition, prolonged uncontrolled blood glucose levels have harmful effects on multiple tissues, including kidney, cardiovascular tissue, eye, neurons, skeletal muscles, and lower limbs (Davoudi and Sobrin, 2015; Sheleme et al., 2020). This disease is associated with several regulatory factors that affect various metabolic pathways in different important organs of the human body. It is evident that obesity plays a crucial role in the etiology of T2D.

According to the data of the National Family Health Survey (2019–2021) obtained from the Global Obesity Observatory, the prevalence of obesity is 22.9% for men and 24% for women, whereas it was 11% and 15% in men and women, respectively, in the 2014–2015 report. These data clearly indicate that the global impact of obesity is increasing at an alarming rate. Thus, this disease requires multifactorial risk reduction strategies and continuous physical and medical care (Altaf et al., 2015).

Plants have been used as medicine since the beginning of civilization. Recently, there is a global thrust in the usage of natural products such as herbs. These natural herbal products contain phytocompounds, which are the chemical compounds synthesized and preserved by plants through their various secondary metabolic pathways. Several of these phytocompounds have medicinal value, are used as crude ingredients in numerous pharmaceuticals and also have a foundational role in modern drug development (Wang et al., 2013; Tran et al., 2020; Jugran et al., 2021). *Gymnema sylvestre* R. Br. is one of the major botanicals used to treat diabetes and obesity in Ayurveda, an Indian traditional system of medicine. Several group of scientists have investigated different formulations of this plant as well as different phytocompounds of which gymnemagenin is the most potent compound used in several antidiabetic Indian traditional AYUSH formulations (Ayurveda, Unani, Siddha, and Homeopathy) as well as nutraceuticals and food supplements (Tiwari et al., 2014). Although numerous scientific studies have been carried out focusing on the hypoglycemic bioactivity of gymnemagenin, there are lacunae in the detailed scientific reports on antiobesity activity. Because obesity and T2D is correlated to a great extent, there is an urgent need to study the role of gymnemagenin in lipid metabolism in correlation with T2D, which could lead to cost-effective targeted phytomedicines with lesser side effects.

The structure and function of adipose tissue are key regulatory factors in lipid metabolism. Adipocyte differentiation, lipid droplet biosynthesis, and lipid droplet size are directly linked with lipolysis, that is, triglyceride metabolism (Albert et al., 2014; Sanjabi et al., 2015). Adipokines, the cell signaling molecules secreted mainly by adipocytes, carry out inter-tissue communication functions, and an imbalance of pro- and anti-inflammatory adipokines contributes to metabolic dysfunction (Ouchi et al., 2011). Lipid transport is associated with muscle insulin sensitivity, which, in turn, regulates glucose transporters and glucose disposal (Hagberg et al., 2012). In this present study, a panel of twelve genes, Fatty acid synthase (*Fasn*), Lipase (*Lipe*), Lipoprotein lipase (*Lpl*), Peroxisome proliferator-activated receptor gamma (*Pparg*), Perilipin 2 (*Plin2*), Cell death-inducing DNA fragmentation factor, alpha subunit-like effector A (*Cidea*), Stearoyl-Coenzyme A desaturase 1 (*Scd1*), Adiponectin (*Adipoq*), Leptin (*Lep*), Chemokine (C-C motif) ligand 2 (*Ccl2*), Fatty acid binding protein 4 (*Fabp4*), Adipocyte solute carrier family 2 (facilitated glucose transporter), and member 4 (*Slc2a4/GLUT4*), which are essential in lipid metabolism and T2D, was selected from literature survey and taken for analysis by *in vitro* and computational approaches to elucidate the role of gymnemagenin in lipid metabolism.

## 2 Materials and methods

### 2.1 Cell culture and treatment

The 3T3L1 mouse embryonic fibroblast cell line was obtained from NCCS, Pune, India. 3T3L1 pre-adipocytes were maintained in Dulbecco’s Modified Eagle’s Medium (DMEM) supplemented with 10% fetal bovine serum (FBS) in a humidified atmosphere containing 5% CO_2_ at 37 °C. Cells were subcultured before the culture reached 70% confluence (Rathinasabapathy et al., 2017).

3T3L1 pre-adipocytes were differentiated into adipocytes following the protocol developed by Rubin, Lai, and Rosen (1977) with minor modifications. Post-confluent pre-adipocytes were supplemented with DMEM (with 10% FBS) media having 1 µM dexamethasone (DEX), 0.5 mM L-methyl-3-isobutylxanthine (MIX) and10 µg/ml insulin. After 3 days, fresh media was added containing DMEM/10% FBS supplemented with 10 µg/ml insulin. The media was changed on alternate days until fully differentiated adipocytes showing well-developed lipid droplets were observed. 3T3L1 pre-adipocytes were plated in 12-well plates and kept for differentiation. Terminally differentiated adipocytes were treated with gymnemagenin in different concentrations (50, 25, 12.5, 6.25, 3.125, 1.56 μM) based on the cell viability data and incubated for 6 h at 37 °C in an incubator with 5% CO_2_. After the incubation, cells were used for RNA isolation and subsequent real-time PCR.

### 2.2 Cytotoxicity assay

3T3L1 cells (5,000 cells/well) were seeded in a 96-well plate and incubated in different concentrations of gymnemagenin (400, 200, 100, 50, 25, 12.5, 6.25 μM) for 24 h. A 10-μL aliquot of MTT (5 mg/ml) was added to each well and incubated for 4 h. Later, formazan crystals were dissolved in 100 μL DMSO, and OD values were taken at 570 nm. OD values were normalized with the blank, and the cell viability percentage was calculated and plotted in a graph.

### 2.3 Determination of triglyceride content by colorimetric estimation

3T3L1 cells were plated in 12-well plates and kept for differentiation. On Day 1 of differentiation, cells were treated with gymnemagenin at different concentrations (50, 25, 12.5, 6.25, 3.125, 1.56 μM). Triglyceride was measured on Day 4 and Day 8 of differentiation using a triglyceride colorimetric assay kit following the manufacturer’s protocol (Elabscience).

### 2.4 RNA preparation and real-time PCR analysis

Total RNA was extracted from cells using TRI reagent (Sigma-Aldrich) following the manufacturer’s protocol with minor modifications. The RNA pellet was dissolved in nuclease-free water. RNA was quantified using Nanodrop. RNA integrity was checked by running the samples in 1% agarose/formaldehyde gel containing 0.5 µg/ml ethidium bromide. cDNAs were synthesized using 1 μg of RNA for each sample using PrimeScript RT Reagent Kit (DSS Takara Bio India), following the manufacturer’s protocol. cDNA was amplified by real-time quantitative PCR (RT PCR) using SYBR green PCR Master Mix (DSS Takara Bio India). Primers of selected genes under this study were procured from Merck (KiCqStart^®^ SYBR^®^ Green Primers) (tabulated in Table 1). RT PCR amplifications were performed on BIORAD CFX Maestro (Version 1.1). Cycle conditions were 95 °C for 30 s, followed by 40 cycles at 95 °C for 15 s and 54°C for 30  s and 72°C for 30 s. mRNA expression was analyzed using the ΔΔCT method and normalized with respect to the expression of the μ-actin using BIORAD CFX Maestro Software (Version 1.1). Amplification of specific transcripts was further confirmed by obtaining dissociation (melting) curve profiles with 1 cycle of 1 min at 95 °C, 30 s at 55 °C, and 30 s at 95 °C (Cho et al., 2009).

**TABLE 1:** Sequences of primers used for RT PCR.

Gene	Primer ID	Primer sequence (5’…3′)
*Fasn*	M_Fas_1	F: TGA​ATG​CCT​CAA​ATC​TTA​GC
		R: TTT​TAG​CTT​CCT​GGA​TTG​TC
*Lipe*	M_Lipe_1	F: AAC​TCC​TTC​CTG​GAA​CTA​AG
		R: CTT​CTT​CAA​GGT​ATC​TGT​GC
*Lpl*	M_Lpl_1	F: GAG​ACT​CAG​AAA​AAG​GTC​ATC
		R: GTC​TTC​AAA​GAA​CTC​AGA​TGC
*Glut4/Slc2a4*	M_Slc2a4_1	F: CAA​TGG​TTG​GGA​AGG​AAA​AG
		R: AAT​GAG​TAT​CTC​ATA​GGA​GGC
*Pparg*	M_Pparg_1	F: AAA​GAC​AAC​GGA​CAA​ATC​AC
		R: GGG​ATA​TTT​TTG​GCA​TAC​TCT​G
*Plin2*	M_Plin2_1	F: ATA​AGC​TCT​ATG​TCT​CGT​GG
		R: GCC​TGA​TCT​TGA​ATG​TTC​TG
*Cidea*	M_Cidea_1	F: GTG​TTA​AGG​AAT​CTG​CTG​AG
		R: CTA​TAA​CAG​AGA​GCA​GGG​TC
*Scd1*	M_Scd1_1	F: GTG​GGG​TAA​TTA​TTT​GTG​ACC
		R: TTT​TTC​CCA​GAC​AGT​ACA​AC
*AdiQ*	M_Adipoq_1	F: CCA​CTT​TCT​CCT​CAT​TTC​TG
		R: CTA​GCT​CTT​CAG​TTG​TAG​TAA​C
*Lep*	M_Lep_1	F: CTT​TGG​TCC​TAT​CTG​TCT​TAT​G
		R: TCT​TGG​ACA​AAC​TCA​GAA​TG
*Ccl2* (*MCP-1*)	M_Ccl2_1	F: CAA​GAT​GAT​CCC​AAT​GAG​TAG
		R: TTG​GTG​ACA​AAA​ACT​ACA​GC
*Fabp4*	M_Fabp4_1	F: GTA​AAT​GGG​GAT​TTG​GTC​AC
		R: TAT​GAT​GCT​CTT​CAC​CTT​CC
*Β-Actin*	M_Actb_1	F: GAT​GTA​TGA​AGG​CTT​TGG​TC
		R: TGT​GCA​CTT​TTA​TTG​GTC​TC

### 2.5 Enrichment analysis of gymnemagenin-regulated targets

Based on the *in vitro* analysis, we subjected 12 genes expressed by gymnemagenin in 3T3L1 cells, *Fabp4*, *Fasn, Lipe, Lpl, Slc2a4, Pparg, Plin2, Cidea, Scd1, Adiq, Lep,* and *Ccl2*, to molecular pathway enrichment analysis. The 12 genes were queried into STRING (Szklarczyk et al., 2016; https://string-db.org/) for *Homo sapiens*, *Mus musculus*, and *Rattus norvegicus* (Khanal et al., 2020). Furthermore, we identified modulated pathways with reference to the Kyoto Encyclopedia of Genes and Genomes (KEGG; https://www.genome.jp/kegg/) pathway database. The pathways associated with diabetes mellitus and obesity were traced (Patil et al., 2020).

### 2.6 Network construction and analysis

The network of gymnemagenin, regulated genes, and modulated pathways in *Homo sapiens*, *Mus musculus*, and *Rattus norvegicus* was constructed by Cytoscape (Shannon et al., 2003) *ver.* 3.6.1 (https://cytoscape.org/). During analysis, the network was treated as direct and analyzed using the edge count topological parameter. To find the hub gene and pathway within the network, the node color and size were set as “low values to bright colors” and “low values to small size,” respectively (Dwivedi et al., 2022).

### 2.7 Molecular docking

Based on the *in vitro* and network analysis, we prioritized Pparg and Fabp4 for molecular docking studies using gymnemagenin and respective standard compounds as ligand molecules. Pioglitazone (Charbonnel, 2009) and BMS-309403 (Lan et al., 2011) compounds were used as standard molecules of Pparg and Fabp4, respectively. PubChem was used to retrieve the 3D structure of phytocompounds. The compounds were prepared using POAP ligand preparation “POAP_lig.bash” script (Samdani and Vetrive, 2018). The structures were minimized by MMFF94 force field using the conjugate gradients algorithm and finally converted into a pdbqt molecule by adding the gasteiger charges and polar hydrogens for further molecular docking study. The 3D x-ray crystallographic structures of Pparg (PDB: 5Y2O) and Fabp4 (PDB: 3JS1) were retrieved from the Protein Data Bank. The PDB ID 5Y2O consisted of missing residues and was remodeled by the SWISS-MODEL (Greenfield and Pietruszko, 1977) web server (https://swissmodel.expasy.org/) using Uniprot ID P37231 as the query sequence and 5Y2O chain A as a template. Furthermore, P2Rank (Krivák and Hoksza, 2018) was employed to retrieve the active site residues information. AutoDock vina was executed using the POAP pipeline (Samdani and Vetrive, 2018; Patil et al., 2020) to perform the molecular docking. During docking, the exhaustiveness was set to 100 and generated nine docked conformations, of which the conformation with the lowest BE and least RMSD was chosen. The interaction between the compound and target was analyzed by BIOVIA Discovery Studio Visualizer 2019 (https://discover.3ds.com/discovery-studio-visualizer-download).

### 2.8 Stability of the docked complexes

To examine the docked complex structural and intermolecular interaction stabilities, an all-atom molecular dynamics (MD) simulation for 100 ns in an explicit solvent was performed. The GROMACS (Van Der Spoel et al., 2005) *ver* 2021.3 (https://www.gromacs.org) package was utilized to run MD simulations using the Amber ff99SB-ildn force field. The topological parameters of the ligands and the whole complex were generated using the AmberTools xleap module (https://ambermd.org/AmberTools.php), and the partial charges of the ligand were generated using an antechamber with a “bcc” charge model. The prepared systems were solvated using the three-site water (TIP3P) model in a rectangular box with 10.0 Å boundary conditions from the protein edges in all directions. The prepared systems were neutralized by adding the required number of counter ions. The steepest descent and conjugate gradient energy minimization methods were used to obtain the near-global state least energy conformations. The systems were equilibrated using “canonical (NVT) and isobaric (NPT)” ensembles for 1 ns. A modified Berendsen thermostat method was used in NVT equilibration to keep the volume and temperature (300 K) constant. A Parrinello–Rahman barostat was used to keep the pressure constant at 1 bar during NPT equilibration. In addition, the particle–mesh Ewald approximation was used with a cut-off value of 1 nm to calculate the long-range electrostatic, van der Waals, and coulomb interactions. Similarly, bond length was constrained using the LINear Constraint Solver method. Finally, the system was subjected to a 100-ns MD production run, and the coordinates were recorded every 2 fs. The trajectories produced were examined using the built-in gromacs tools. RMSD, RMSF, R*g*, SASA, and H-bond were used to investigate the stability and fluctuations of ligand-protein interactions using MD simulation.

### 2.9 Molecular mechanics Poisson–Boltzmann surface area (MM-PBSA): Investigation of binding affinity

In MD simulations and thermodynamic calculations, the relative binding energies were calculated using the MM-PBSA method using the “g_mmpbsa” tool (Kumari and Kumar, 2014). The parameters from past research were considered while calculating the binding energy (Bhandare et al., 2019; Dwivedi et al., 2022; Khanal et al., 2022). The binding energy was determined throughout the steady trajectory observed between 50 and 100 ns using 50 representative snapshots. Binding free energy obtained from MM-PBSA was represented in kJ/mol units.

### 2.10 Analysis of principal component

Principal component analysis (PCA) investigates molecular motion using MD trajectories. The “least square fit” to the reference structure is used to eliminate the molecule’s translational and rotational motion. A set of eigenvectors that reflect the motion of the molecule is produced by diagonalizing a covariance matrix generated by a linear transformation of cartesian coordinate space. The energy contribution of each eigenvector to the motion is shown by the eigenvalue associated with that eigenvector. The “time-dependent motions” that the components carry out in a certain vibrational mode are demonstrated by projecting the trajectory onto a particular eigenvector. The atomic vibrational components’ contribution to this form of coordinated motion is shown by the projection’s temporal average. Using the built-in gromacs utility “g_covar,” the eigenvectors and eigenvalues of the trajectory were produced by computing and diagonalizing the covariance matrix. Additionally, the eigenvectors were examined and shown using the “g_anaeig” tool (Amadei et al., 1993; Van Aalten et al., 1995; Amadei et al., 1996; Bhandare and Ramaswamy, 2018). The least squares fit, gromacs inbuilt utility g_covar, and g_anaeig tools were used for PCA.

### 2.11 Statistical analysis

All experiments were done in triplicate. All statistical analyses were done by analyzing two-variable data with a simple *t*-test and using a one-way analysis of variance (ANOVA). The value of *p* < 0.05 was considered statistically significant. The network was analyzed by the “Edge count” topological parameter. The docking score was represented in kcal/mol units.

## 3 Results

### 3.1 Cytotoxicity assay

**FIGURE 1:**
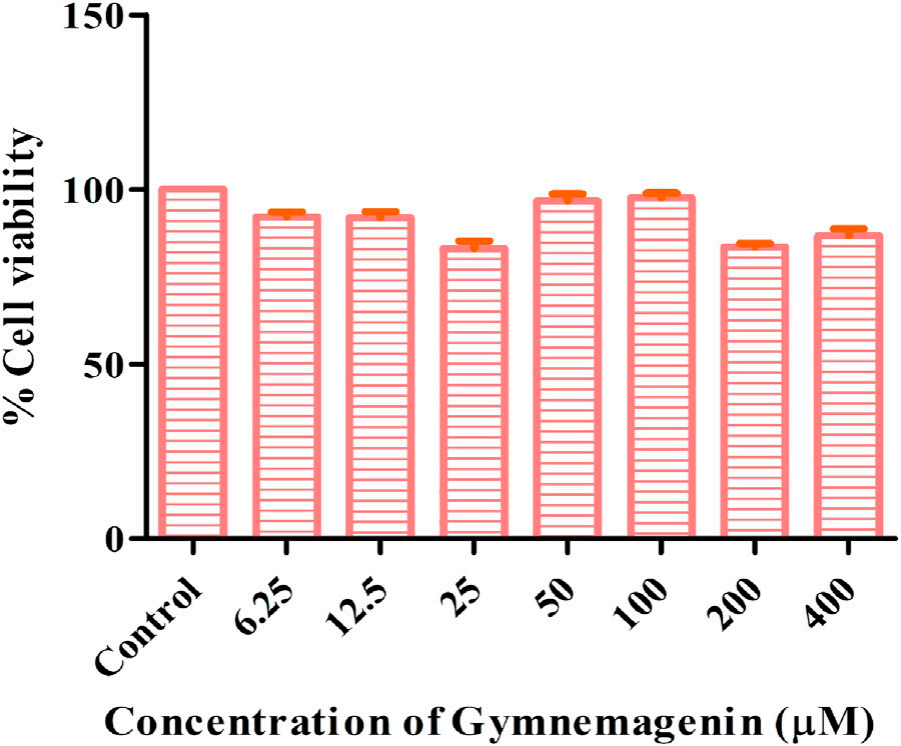
Determination of cytotoxic effects of gymnemagenin in 3T3L1 cells. 3T3L1 cell viability upon gymnemagenin treatment was assessed by MTT assay.

The cytotoxic effects of gymnemagenin were tested in 3T3L1 cells by MTT assay. Only metabolically viable cells convert tetrazolium salts to formazan dye by cellular enzymes. Thus, the amount of formazan dye formed directly correlates to the number of viable cells in the culture and can be measured in a spectrophotometer, whereas cells exposed to toxins will have decreased activity. At the highest concentration of gymnemagenin, 86.8% of the cells were viable (Figure 1).

### 3.2 Gymnemagenin promotes triglyceride metabolism in 3T3L1 cells

**FIGURE 2:**
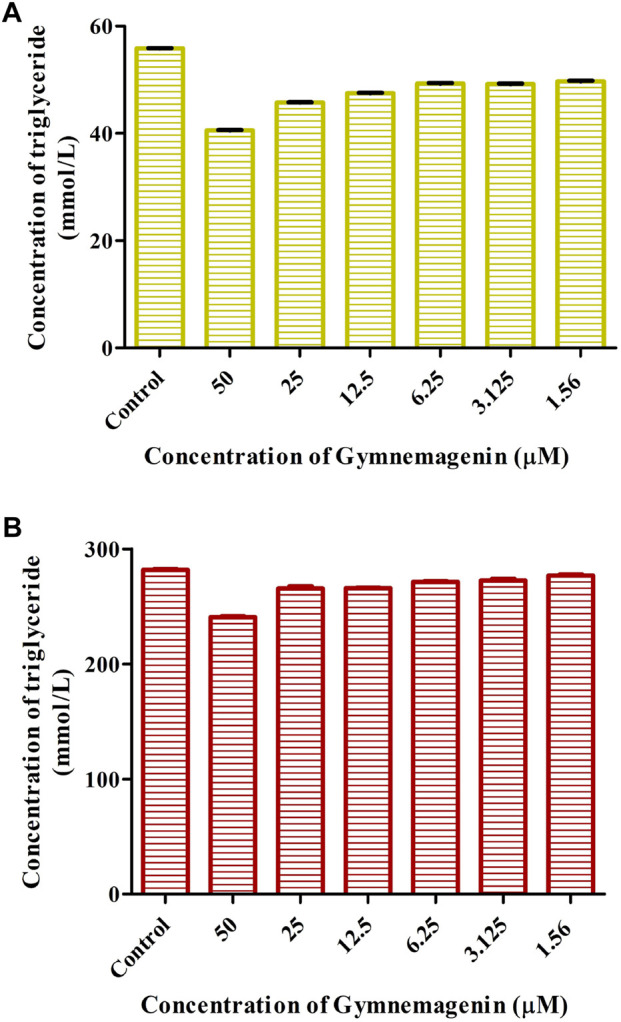
Effect of gymnemagenin on triglyceride content of adipocytes. **(A)** Triglyceride content after gymnemagenin treatment on the 4th day of differentiation in 3T3L1 adipocytes. **(B)** Triglyceride content after gymnemagenin treatment on the 8th day of differentiation in 3T3L1 adipocytes.

To examine the effects of gymnemagenin on triglyceride metabolism, the triglyceride content of 3T3L1 adipocytes differentiated from gymnemagenin untreated and treated pre-adipocytes was measured by colorimetric estimation. The triglyceride content of the gymnemagenin-treated group was less than that of the untreated group on the fourth day of the differentiation stage (Figure 2A) as well as on the eighth day of the differentiation stage (Figure 2B), depicting hydrolysis of more triglyceride by *Lpl* into glycerol and free fatty acids due to induction of gymnemagenin. This was further confirmed by gene expression studies.

### 3.3 Gymnemagenin improves lipid metabolism in 3T3L1 cells

To elucidate the effect of gymnemagenin on lipid metabolism, we examined the expression of 12 genes with and without gymnemagenin treatment in 3T3L1 adipocytes. Twelve genes were categorized into five categories: lipolysis or triglyceride metabolism, adipocyte differentiation, adipokine/adipocyte function, lipid transport, and insulin signaling. Table 2 describes the function of selected genes and their expression pattern in obesity and T2D. In addition, the expression pattern of those genes after gymnemagenin treatment has also been tabulated in Table 2.

**TABLE 2:** Effect of gymnemagenin on the 12 gene panel of lipid metabolism and T2D.

Category	Gene symbol	Gene full name	Specific function	Expression pattern in obesity/T2D	Expression pattern after gymnemagenin treatment	References
Triglyceride metabolism	Fasn	Fatty acid synthase	Lipolytic enzyme: Lipase catalyzes the hydrolysis of triglyceride into fatty acid and glycerol.	↑	No significant change	Berndt et al. (2007)
	Lipe	Lipase, hormone sensitive	Lipolytic enzyme: Lipase catalyzes the hydrolysis of diglyceride into fatty acid and glycerol.	↓	↑	Albert et al. (2014)
	Lpl	Lipoprotein lipase	Lipolytic enzyme: Catalyses the hydrolysis of triglycerides from circulating chylomicrons and very low-density lipoproteins (VLDL) and thereby plays an important role in lipid clearance from the bloodstream, lipid utilization and storage.	↓	↑	Kase et al. (2015)
Adipocyte differentiation	Pparg	Peroxisome proliferator-activated receptor gamma	Key regulator of adipocyte differentiation and glucose homeostasis. Transcriptionally regulates Plin2 and Cidea and has an important role in lipid droplet formation and size.	↓	↑	Albert et al. (2014)
	Plin2	Perilipin 2	A lipid droplet scaffold protein involved in the efflux of lipolytic enzymes like Lipe. Has a role in adipocyte differentiation and maintenance.	↓	↑	Minnaard et al. (2009)
	Cidea	Cell death-inducing DNA fragmentation factor, alpha subunit-like effector A	Lipid droplet size: negatively regulates lipolysis and promotes increased lipid droplet size. Binds to lipid droplets and regulates their enlargement, thereby restricting lipolysis and favoring storage.	↓	↑	Albert et al. (2014)
	Scd1	Stearoyl-Coenzyme A desaturase 1	Regulates fatty acid composition in lipid droplet and ultimately, the size of adipocytes. It is PPARγ dependent. Plays an important role in lipid biosynthesis.	↓	↑	Albert et al. (2014)
Adipokine/Adipocyte function	Adipoq	Adiponectin, C1Q and collagen domain containing	Important adipokine involved in the control of fat metabolism and insulin sensitivity.	↓	↑	Kandasamy et al. (2012)
	Lep	Leptin	Increases phagocytosis by macrophages and enhances the secretion of pro-inflammatory mediators. Plays a pro-inflammatory role, in synergy with IL1B, by inducing NOS2, which promotes the production of IL6, IL8, etc.	↓	↑	Al-Harithy and Alomari (2021)
	Ccl2	Chemokine (C-C motif) ligand 2	Large adipocytes release it. Acts as a ligand for the C-C chemokine receptor CCR2.	↓	↑	Kandasamy et al. (2012)
Lipid transport	FABP4	fatty acid binding protein 4, adipocyte	Lipid transport protein in adipocytes.	↑	No significant change	Furuhashi et al. (2015)
Insulin signaling	Slc2a4 (GLUT4)	solute carrier family 2 (facilitated glucose transporter), member 4	Insulin-regulated facilitative glucose transporter, which plays a key role in the removal of glucose from circulation.	↓	No significant change	Michael et al. (2001)

**FIGURE 3:**
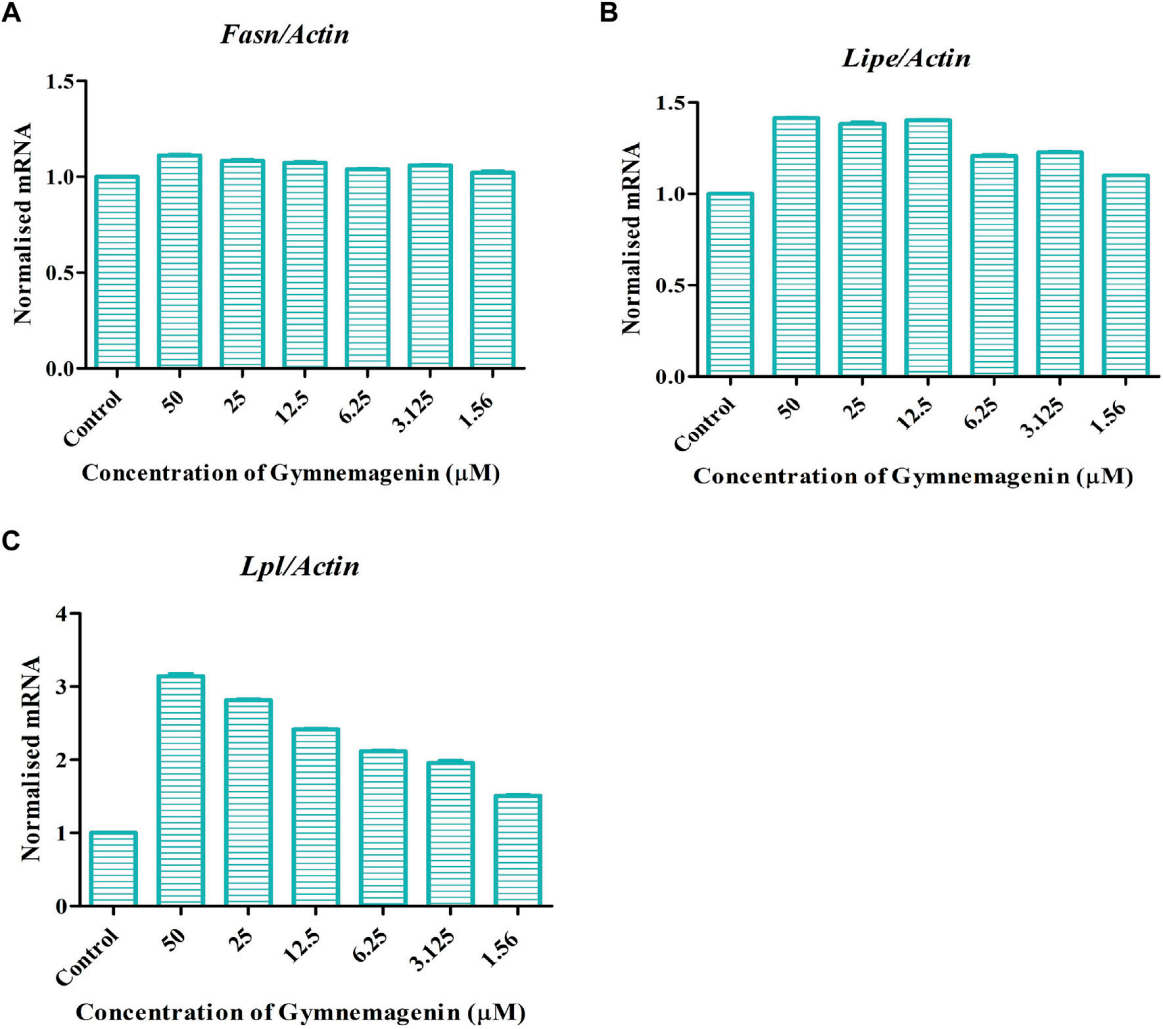
Effect of gymnemagenin treatment on lipase enzyme coding genes expressed in 3T3L1 adipocytes. Data represent the mean ± SE. *n* = 3 per group. **(A)** Gymnemagenin showed no effect on the expression of Fasn gene, **(B)** Gymnemagenin showed no effect on the expression of Lipe in treated cells, **(C)** Gymnemagenin increased the expression of Lpl gene in the treated cells.

Three genes, *Fasn*, *Lipe* and *Lpl*, are key regulators of lipolysis or triglyceride metabolism. Gymnemagenin upregulated the expression pattern of *Lipe* and *Lpl* by 1.4 and threefold, respectively. However, the expression of *Fasn* was unaltered due to gymnemagenin (Figure 3).

**FIGURE 4:**
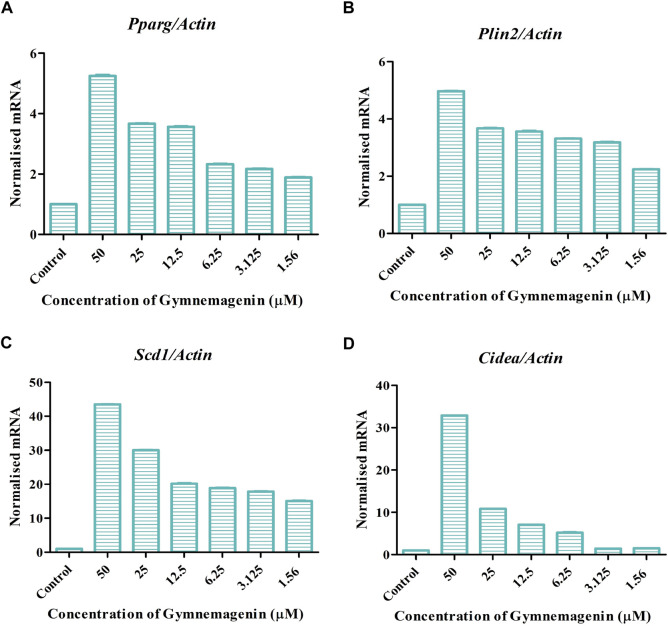
Effect of gymnemagenin on genes actively involved in adipogenesis expressed in 3T3L1 adipocytes. **(A)** Gymnemagenin increased the expression of *Pparg* in treated cells more than in control cells. **(B)** Gymnemagenin escalated the expression of *Plin2*. **(C)** Gymnemagenin increased the expression of *Scd1*. **(D)** Gymnemagenin increased the expression of *Cidea*. Data represent the mean ± SE. *n* = 3 per group.

Adipocyte differentiation (adipogenesis) is highly regulated by four genes, *Pparg*, *Plin2*, *Cidea*, and *Scd1* (Albert et al., 2014). It is reported that in obesity and T2D disease conditions, the expression of these genes was decreased (Minnaard et al., 2009). In our study, it was found that gymnemagenin upregulated the expression of *Pparg, Plin2, Cidea* and *Scd1* (Figure 4).

**FIGURE 5:**
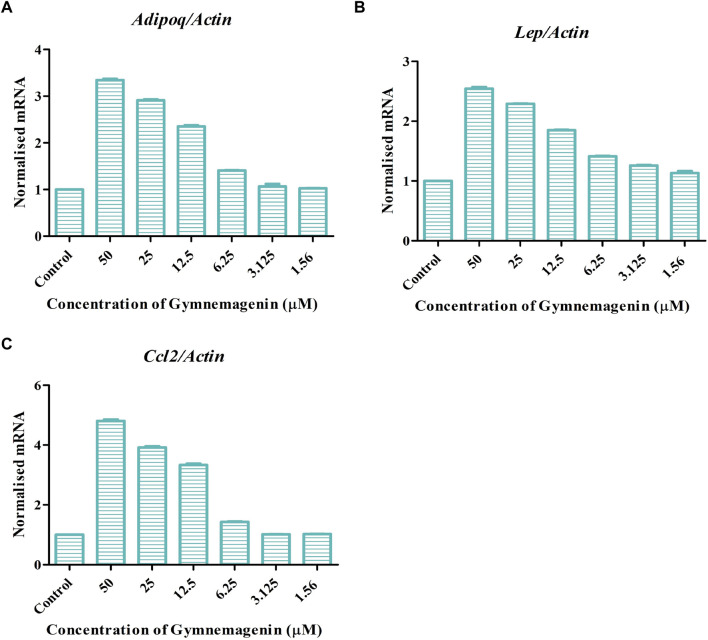
Effect of gymnemagenin treatment on expression of adipokines secreted by adipocytes. **(A)** Gymnemagenin increased the expression of *Adipoq* in treated cells more than in control cells. **(B)** Gymnemagenin increased the expression of *Lep*. **(C)** Gymnemagenin increased the expression of *Ccl2*. Data represent the mean ± SE. *n* = 3 per group.

Adipokines are cell-signaling molecules produced by the adipose tissue that play functional roles in obesity, energy metabolism and inflammation (Kandasamy et al., 2012). *Adipoq*, *Lep*, and *Ccl2* are vital adipokines that have a crucial role in the maintenance of body mass, immune responses, fatty acid storage, and metabolic balance. At the onset of obesity, all three adipokines showed decreased gene expression. Induction of gymnemagenin resulted in the upregulation of all three genes in 3T3L1 adipocytes (Figure 5).

**FIGURE 6:**
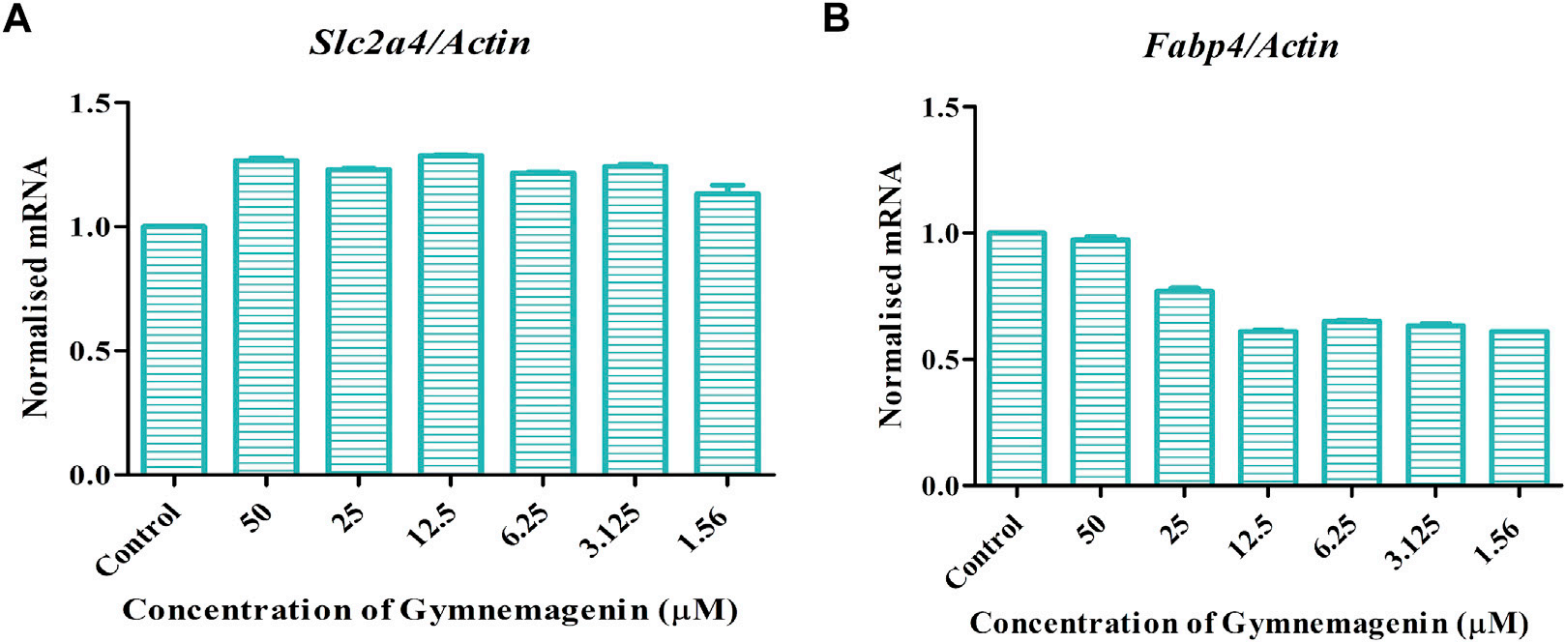
**(A)** Effect of gymnemagenin treatment on expression of *Slc2a4.* **(B)** Expression of *Fabp4* in adipocytes was unchanged after gymnemagenin treatment. Data represent the mean ± SE. *n* = 3 per group.

Fabp4 is an intracellular lipid chaperone that regulates lipid trafficking and lipolysis in adipocytes. The present study showed that gymnemagenin had no effect on the expression of *Fabp4* in 3T3L1 adipocytes (Figure 6A). Solute carrier family 2 (facilitated glucose transporter) member 4 (Slc2a4/GLUT4) is a facilitative glucose transporter regulated by insulin. The data showed that gymnemagenin had no significant effect on the expression of *Slc2a4* (Figure 6B).

### 3.4 Gymnemagenin-modulated gene pathways enrichment analysis

**FIGURE 7:**
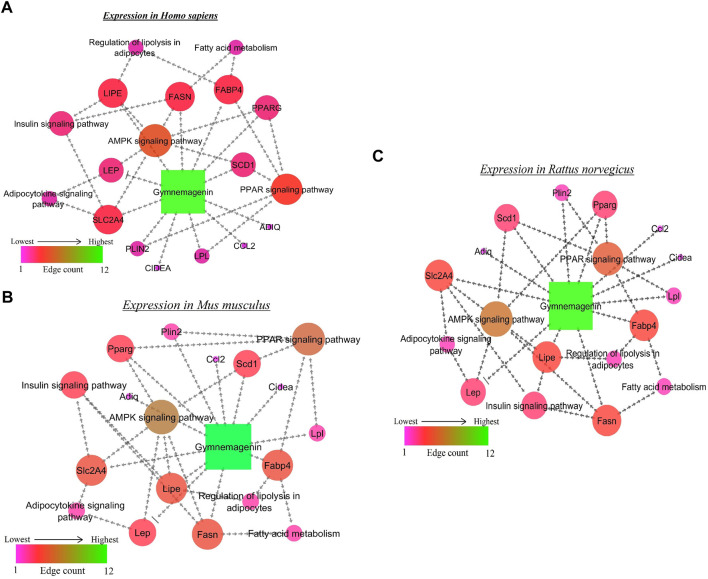
Network representation of gymnemagenin-modulated genes and pathways in **(A)** *Homo sapiens*, **(B)** Mus musculus, and **(C)** Rattus norvegicus. In the network interaction, the node with the higher edge is represented with green color, which gradually decreases to pink.

From the *in vitro* study, twelve genes were targeted by gymnemagenin. Among them, 11 were upregulated, and one was downregulated. Gene set enrichment of twelve genes was predicted to modulate six molecular pathways: AMPK, PPAR, insulin, Adipocytokine signalling and Fatty acid metabolism are different and considered as two pathways, and regulation of lipolysis in adipocytes in *Homo sapiens*, *Mus musculus*, and *Rattus norvegicus.* The pathway strength and FDR value were found to be different in the three species, and all six pathways were found to be similar. Figure 7 summarizes the network representation of gymnemagenin, its modulated targets, and pathways. Table 3 summarizes the gene set enrichment of individual pathways of each species**.**

**TABLE 3:** Gymnemagenin-modulated 12-gene enrichment analysis in *Mus musculus*, *Homo sapiens,* and *Rattus norvegicus*.

Organism	KEGG ID	Pathway	Gene count	Strength	FDR	Gene set within the pathway
*Mus musculus*	mmu04152	AMPK signaling pathway	6	1.95	1.06E−08	*Pparg, Lipe, Slc2a4, Scd1, Fasn, Lep*
	mmu03320	PPAR signaling pathway	5	2.02	1.45E−07	*Pparg, Plin2, Lpl, Fabp4, Scd1*
	mmu04910	Insulin signaling pathway	3	1.62	0.0054	*Lipe, Slc2a4, Fasn*
	mmu01212	Fatty acid metabolism	2	1.78	0.0353	*Scd1, Fasn*
	mmu04923	Regulation of lipolysis in adipocytes	2	1.82	0.0353	*Lipe, Fabp4*
	mmu04920	Adipocytokine signaling pathway	2	1.71	0.0386	*Slc2a4, Lep*
*Homo sapiens*	hsa04152	AMPK signaling pathway	6	1.95	9.53E−09	*LIPE, PPARG, FASN, LEP, SLC2A4, SCD1*
	hsa03320	PPAR signaling pathway	5	2.07	7.64E−08	*FABP4, PLIN2, PPARG, LPL, SCD1*
	hsa04910	Insulin signaling pathway	3	1.6	0.0058	*LIPE, FASN, SLC2A4*
	hsa01212	Fatty acid metabolism	2	1.82	0.0365	*FASN, SCD1*
	hsa04923	Regulation of lipolysis in adipocytes	2	1.82	0.0365	*LIPE, FABP4*
	hsa04920	Adipocytokine signaling pathway	2	1.71	0.0391	*LEP, SLC2A4*
*Rattus norvegicu*	rno04152	AMPK signaling pathway	6	1.98	7.59E−09	*Pparg, Scd1, Slc2a4, Lipe, Fasn, Lep*
	rno03320	PPAR signaling pathway	5	2.08	7.33E−08	*Plin2, Pparg, Fabp4, Lpl, Scd1*
	rno04910	Insulin signaling pathway	3	1.66	0.004	*Slc2a4, Lipe, Fasn*
	rno01212	Fatty acid metabolism	2	1.82	0.032	*Scd1, Fasn*
	rno04923	Regulation of lipolysis in adipocytes	2	1.85	0.032	*Fabp4, Lipe*
	rno04920	Adipocytokine signaling pathway	2	1.75	0.0333	*Slc2a4, Lep*

### 3.5 Molecular docking

**FIGURE 8:**
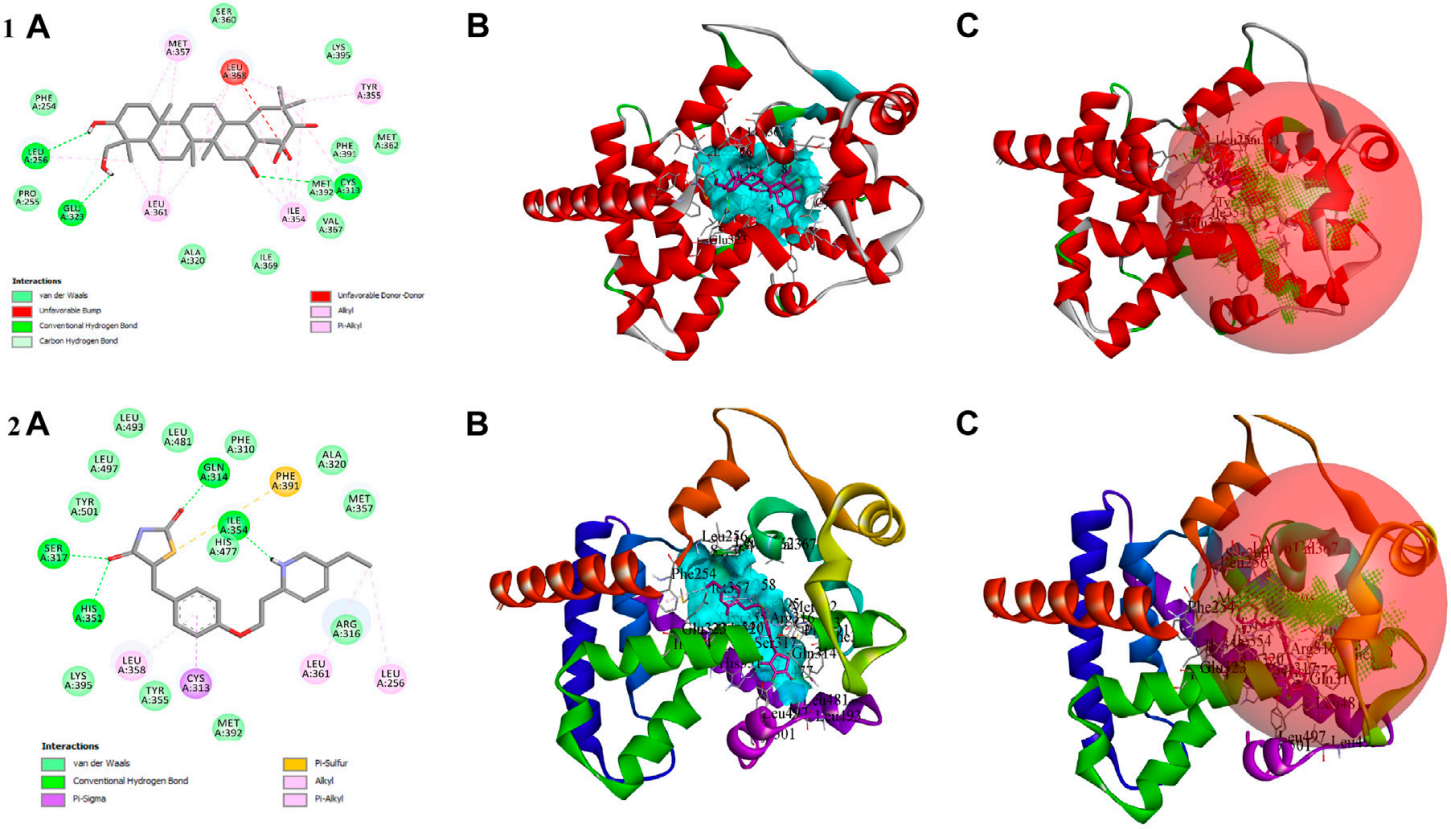
Shows **(1A)** the 2D interaction of the binding mode of gymnemagenin with Pparg, **(1B)** 3D binding mode of gymnemagenin with Pparg, **(1C)** active site representation of gymnemagenin with Pparg, **(2A)** the 2D interaction of binding mode of pioglitazone with Pparg, **(2B)** 3D binding mode of pioglitazone with Pparg, and **(2C)** active site representation of pioglitazone with Pparg.

In the *in vitro* study, gymnemagenin significantly upregulated *Pparg* compared to other genes, and it also transcriptionally regulated other genes. Hence, we initially prioritized performing molecular docking of gymnemagenin with Pparg. Additionally, we also selected another protein, Fabp4, which was found to be involved in the Pparg signaling pathway but not significantly modulated by gymnemagenin. In the remodeled Pparg structure, about 99.1 and 8.1% of residues were in the most favored and additionally allowed region. The RMSD of the template and model was found to be 0.064 (Supplementary Figure S1). The active site residue numbers of Pparg are Phe254, Pro255, Leu256, Ile277, Leu283, Gly286, Glu287, Phe292, Ile295, Ile309, Phe310, Gly312, Cys313, Gln314, Arg316, Ser317, Ala320, Glu323, Ile354, Tyr355, Met357, Leu358, Leu361, val367, Leu368, Ile369, Ser370, Glu371, Met376, Leu381, Phe391, Met392, His477, Leu481, Leu497, and Tyr501. The active site residue numbers of Fabp4 are Phe17, Tyr20, Met21, Val26, Ala34, Pro39, Met41, Ile52, Ser54, Ser56, Phe58, Lys59, Thr61, Ile63, Glu73, Thr75, Ala76, Asp77, Gln94, Gln96, Ile105, Arg107, Val116, Cys118, Arg127, and Tyr129. Docking of compounds with Pparg revealed that a standard molecule pioglitazone scored the lowest BE of −8.7 kcal/mol *via* forming four hydrogen bond interactions with Ser317, His351, Gln314, and Ile354 and five non-hydrogen bond interactions with Cys313, Leu358, Met357, and Phe254. Meanwhile, gymnemagenin scored the lowest BE of −9.1 kcal/mol *via* forming three hydrogen bonds with Leu256, Glu323, and Cys313 and 18 non-hydrogen bonds with Met357 (2), Leu256, Glu323, Leu361 (4), Ile354 (4), Cys313, Tyr355, and Leu358 (4). Figure 8 represents the interaction of pioglitazone and gymnemagenin with Pparg.

**FIGURE 9:**
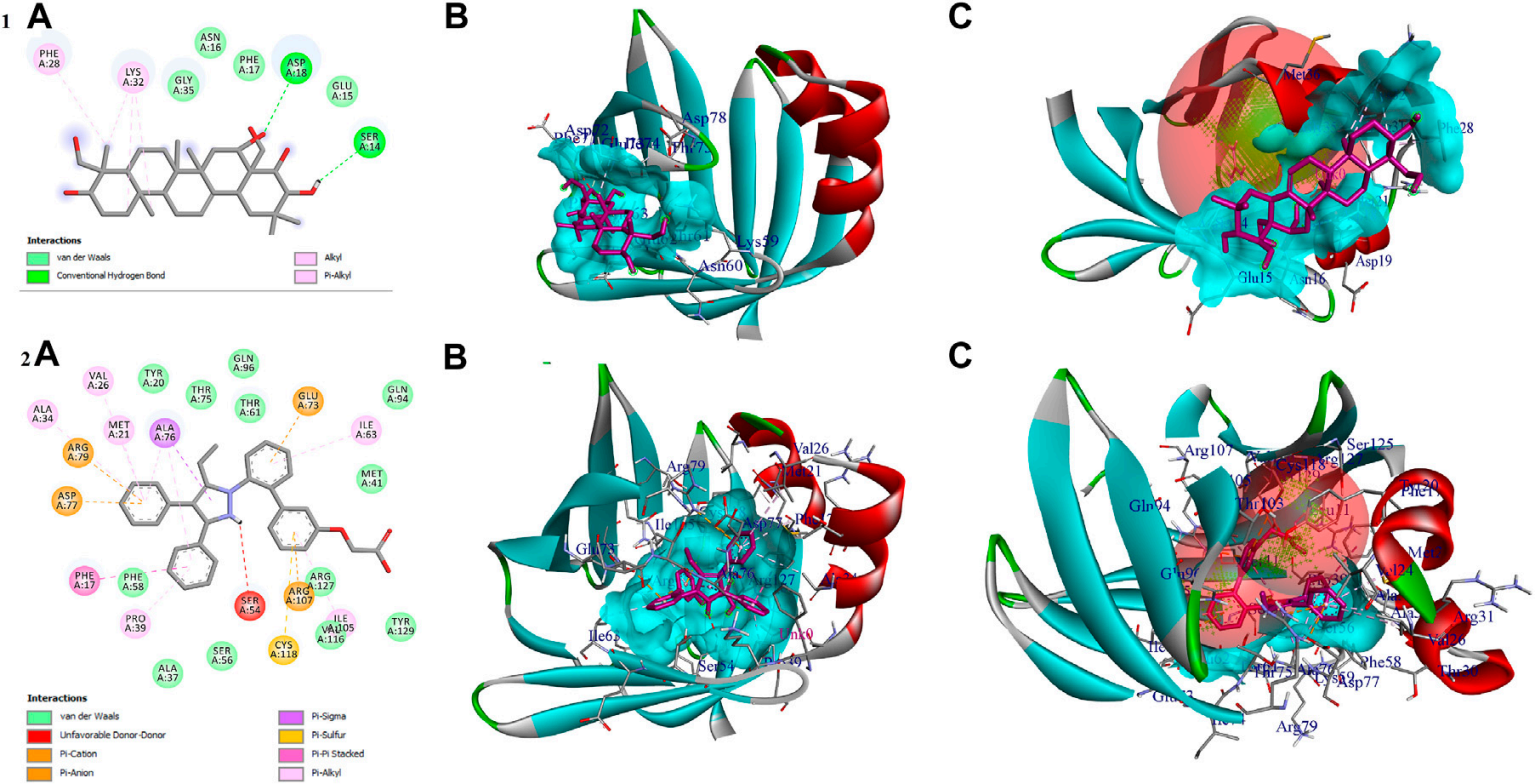
Shows **(1A)** the 2D interaction of the binding mode of gymnemagenin with Fabp4, **(1B)** 3D binding mode of gymnemagenin with Fabp4, **(1C)** active site representation of gymnemagenin with Fabp4, **(2A)** the 2D interaction of binding mode of BMS-309403 with Fabp4, **(2B)** 3D binding mode of BMS-309403 with Fabp4, and **(2C)** active site representation of BMS-309403 with Fabp4.

The compound gymnemagenin scored the lowest BE of −6.1 kcal/mol *via* forming two hydrogen bond interactions with Asp18 and Ser14 and four non-hydrogen bonds with Phe28 and Lys32 (3). Gymnemagenin was found to bind adjacent to the active site (Figure 9), whereas the standard molecule BMS-309403 scored the lowest BE of −10.1 kcal/mol *via* forming fifteen non-hydrogen bonds: Ala34, Val26, Arg79, Asp77, Met21, Ala76 (3), Glu73, Ile63, Ile105, Arg107, Cys118, Pro39, and Phe17. Among these interactions, 14 interactions were with active site residues except Arg79.

### 3.6 Molecular dynamics

#### 3.6.1 Stability of gymnemagenin and pioglitazone with pparg

**FIGURE 10:**
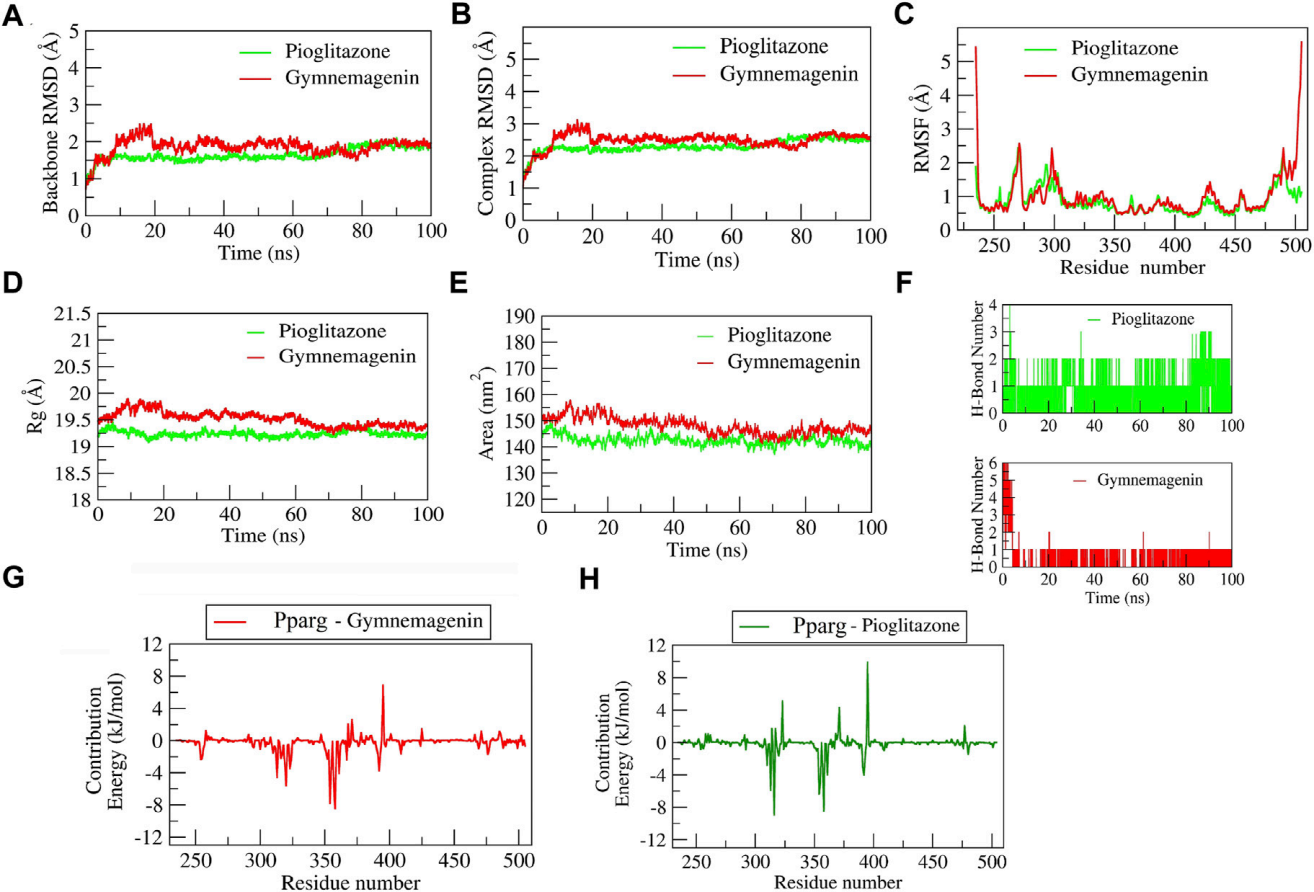
Parameters describing gymnemagenin and pioglitazone–Pparg complex structural stabilities. **(A)** RMSD of backbone, **(B)** RMSD of complex, **(C)** RMSF, **(D)** Rg, **(E)** SASA, **(F)** number of H-bond interactions, and **(G,H)** per-residue contribution energy plot highlighting the importance of the binding pocket residues in stable complex formation.

The complex “gymnemagenin-Pparg” and “pioglitazone-Pparg” showed stable dynamics during the 100 ns of simulation after a 20-ns equilibration period (Figure 10). The average backbone RMSDs for gymnemagenin and pioglitazone were 1.86 Å and 1.64 Å, and the complex RMSDs were 2.48 Å and 2.30 Å, respectively. The N- and C-terminal residues showed maximum residual fluctuations (∼5 Å) in the gymnemagenin-Pparg complex compared to pioglitazone-Pparg. However, the residues engaged in the stable and conserved non-bonded interactions (Leu256, Ser317, His351, Ile354, Phe391, Gln314, Glu323, Cys313, Met357, Tyr355, Leu361, Tyr355) showed relatively smaller fluctuations (∼2.5 Å) in both complexes. In the gymnemagenin-Pparg complex, a gradual decrease in the Rg value was observed, and stable complex formation was seen after ∼70 ns. After 70 ns, the gymnemagenin-PPARG and pioglitazone-Pparg complex average Rg values were found to be ∼19.25 Å. In addition, it was observed that the initial and final average surface areas occupied by gymnemagenin and pioglitazone with Pparg were 150.59 and 145.90 nm^2^ and 143.51 nm^2^ and 136.88 nm^2^, respectively. The average surface areas occupied by gymnemagenin and the pioglitazone complex were 148.06 nm^2^ and 142.49 nm^2^, respectively, which represents higher flexibility of the binding pocket in the presence of gymnemagenin. Supplementary Movies S1, S2 represent the detailed binding mode and stable dynamics of gymnemagenin and pioglitazone with Pparg. Gymnemagenin formed six stable H-bonds, of which one was consistent throughout the simulations, whereas pioglitazone formed four, of which one was consistent. The estimated relative binding energy of the complex gymnemagenin-Pparg was −177.94 ± 12.82 kJ/mol, and the van der Waal, electrostatic, polar solvation, and SASA energies were −267.87 ± 9.517, −12.35 ± 5.99, 128.34 ± 11.65, and −26.07 ± 0.602 kJ/mol, respectively. Similarly, the estimated relative binding energy of the pioglitazone-PPARG complex was −109 ± 19.17 kJ/mol, and the van der Waal, electrostatic, polar solvation, and SASA energies were −209.099 ± 11.79, −31.97 ± 8.98, 153.63 ± 11.99, and −21.71 ± 0.896 kJ/mol. Furthermore, the contribution of the active residue in complex formation was calculated. The per residue contribution energy of the gymnemagenin-Pparg complex revealed Cys313, Arg316, Ile354, Tyr355, Met357, Leu358, and Leu361 residues from the binding pocket contribute significantly in forming a stable complex *via* scoring contribution energies of −5.89, −8.94, −6.35, −4.82, −2.70, −8.43, and −3.98 kJ/mol, respectively. Similarly, the per residue contribution energy of the pioglitazone-Pparg complex revealed Cys313, Arg316, Ser317, Glu319, Ala320, Glu323, Ile324, Ile354, Tyr355, Met357, Leu358, Leu361, Val367, and Met392 residues from the binding pocket contribute significantly to forming stable complex *via* scoring contribution energies of −4.60, −2.19, −1.73, −2.93, −5.59, −3.46, −2.23, −7.81, −3.45, −5.93, −8.44, −4.69, −2.40, and −3.75 kJ/mol, respectively.

#### 3.6.2 Stability of gymnemagenin and BMS-309403 with Fabp4

**FIGURE 11:**
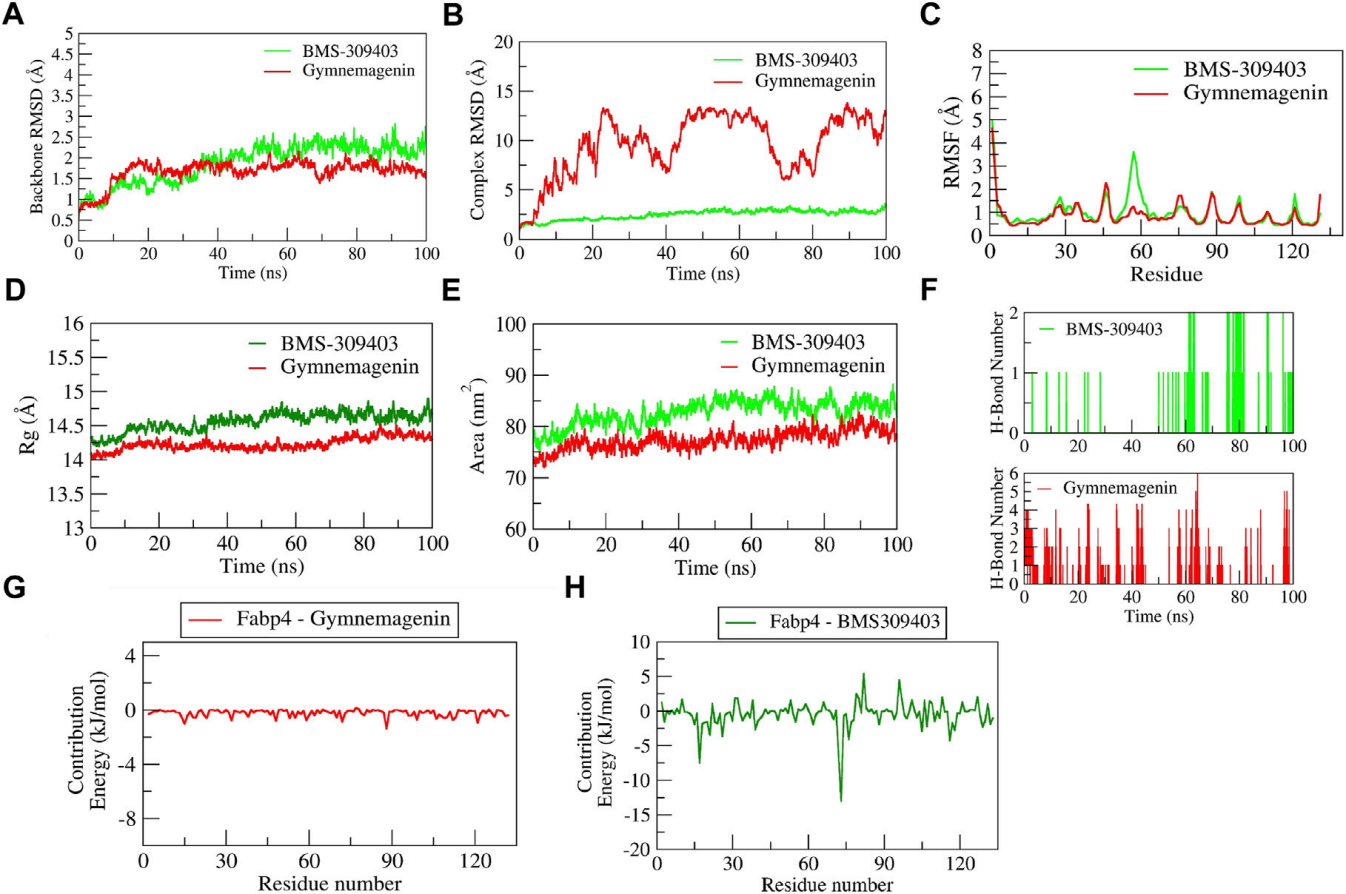
Parameters describing gymnemagenin and BMS-309403–Fabp4 complex structural stabilities. **(A)** RMSD of backbone, **(B)** RMSD of complex, **(C)** RMSF, **(D)** Rg, **(E)** SASA, **(F)** number of H-bond interactions, **(G,H)** per-residue contribution energy plot highlighting the importance of the binding pocket residues in stable complex formation.

The complex BMS-309403-Fabp4 showed stable dynamics during the 100 ns of simulation after a 30-ns equilibration period compared to the gymnemagenin-Fabp4 complex (Figure 11). The average backbone RMSDs for gymnemagenin and BMS-309403 were 1.67 Å and 1.86 Å, and the complex RMSDs were 9.6 Å and 2.48 Å, respectively. Gymnemagenin was found to be unstable with Fabp4 as it was found to move out of the binding pocket (Supplementary Movie S3). The N-terminal residues showed maximum residual fluctuations (∼5 Å) in both complexes. However, the residues engaged in the stable and conserved non-bonded interactions (Glu73, Arg79, Asp77, Phe17, Arg107, Cys118, Ile105, Ile63, Ala34, Val26, Met21, Ala76, Pro39, Ala76) with BMS-309403 showed relatively smaller fluctuations (∼2.0 Å). In gymnemagenin and the BMS-309403 complex, the protein Rg was found to be stable at ∼14.5 and 14.15 Å, respectively. Similarly, the surface area was also found to be stable for gymnemagenin (∼73 nm^2^) and BMS-309403 (83 nm^2^). Supplementary Movies S3, S4 represent the detailed binding mode and stable dynamics of gymnemagenin and BMS-309403 with Fabp4. Gymnemagenin formed six stable H-bonds with zero consistent interaction throughout the simulation. In contrast, BMS-309403 formed two bonds, of which one was consistent. The estimated relative binding energy of the complex gymnemagenin-Fabp4 was −25.406 ± 30.581 kJ/mol, and the van der Waal, electrostatic, polar solvation, and SASA energies were −0.007 ± 0.01, −0.25 ± 0.31, −25.38 ± 30.251, and 0.089 ± 1.137 kJ/mol. Similarly, the estimated relative binding energy of the complex BMS-309403-FABP4 was −21.067 ± 15.68 kJ/mol, and the van der Waal, electrostatic, polar solvation, and SASA energies were −190 ± 13.82, 64.69 ± 24.71, −126.996 ± 28.67, and −22.363 ± 1.412 kJ/mol. Furthermore, the contributions of the active residues in complex formation were calculated. The per residue contribution energy of the gymnemagenin-Fabp4 complex revealed no significant residues from the binding pocket to contribute to forming a stable complex. The contribution energies of the binding pocket residues were less than 0.4 kJ/mol. Similarly, the per-residue contribution energy of BMS-309403-Fabp4 complex revealed Phe17, Met21, Val26, Pro39, Asp72, and Glu73 residues from the binding pocket contributed significantly in forming stable complex *via* scoring contribution energies of −7.50, −3.45, −3.64, −2.70, −6.52, and −13.05 kJ/mol, respectively.

### 3.7 Principal component analysis

**FIGURE 12:**
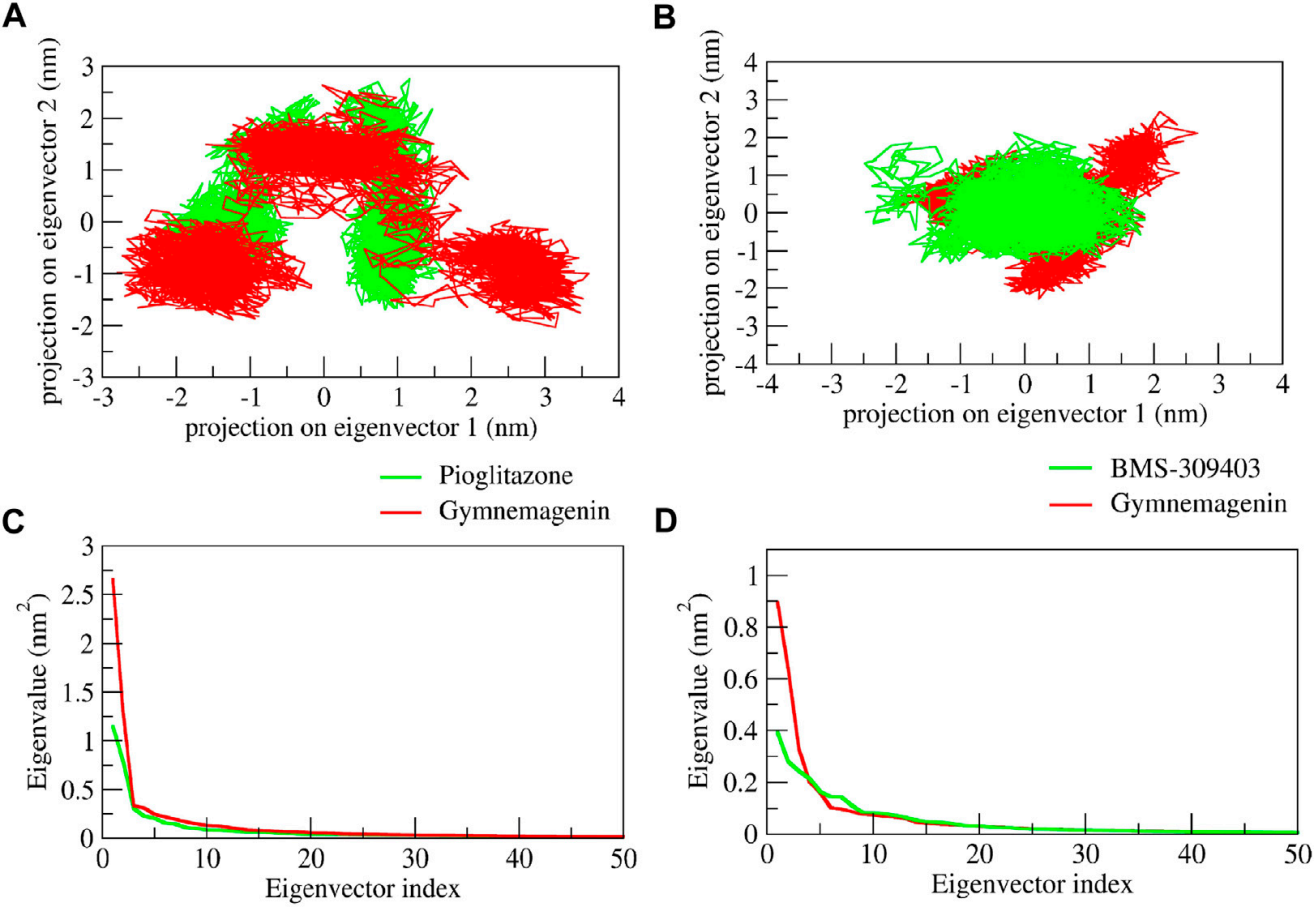
Principal component analysis of protein-ligand complexes: the collective motion of gymnemagenin (red) and standard (green) with **(A)** Pparg and **(B)** Fabp4 using projections of MD trajectories on two eigenvectors corresponding to the first two principal components. The first 50 eigenvectors were plotted versus the eigenvalue for gymnemagenin (red) and standard (green) with **(C)** Pparg and **(D)** Fabp4.

We used PCA to investigate the diversity and structural flexibility that result from the stable trajectory generated from 100-ns MD simulations. The first 50 eigenvectors/principal components capture the most collective motion. As a result, we carefully investigated the first two principal components (PCs). The first two eigenvectors’ 2D projection is shown in Figure 12. In the pioglitazone-Pparg complex, it was observed that pioglitazone showed less diversity of conformations during the simulations, whereas gymnemagenin in complex with Pparg showed a similar diversity of conformations with slight flexibility during simulation. However, gymnemagenin showed a larger diversity of conformations of Fabp4 during simulation compared to the standard molecule BMS-309403. This reveals that gymnemagenin forms a stable complex with Pparg but not with Fabp4.

## 4 Discussion

Obesity is one of the most vital modifiable risk factors for the prevention of T2D. Adipose tissue is an important endocrine organ. Its metabolic abnormalities are associated with obesity and result in dysregulation of multiple metabolic pathways in other tissues. Indeed, the functional and structural balance of white adipose tissue and lipids is essential for the prevention of metabolic diseases and associated complications. Several groups of scientists have worked on the functions of adipose tissue and the genetic understanding of associated lipid metabolism (Berndt et al., 2007; Kandasamy et al., 2012; Albert et al., 2014).

Lipids are stored as triglycerides or triacylglycerols in lipid droplets in skeletal muscles. It is reported that an increased triglyceride level is associated with lipotoxic intermediate metabolites that inhibit insulin signaling and, ultimately, insulin resistance and obesity. Thus, intra-myocellular dynamics of lipid and lipid turnover have a significant impact on obesity and insulin resistance (Kase et al., 2015). The drug therapies for hypertriglyceridemia are still under development. In the present study, we found that gymnemagenin treatment decreased the triglyceride content of 3T3l1 adipocytes. The further examination of the effect of gymnemagenin on the gene expression of three lipases, *Fasn*, *Lipe*, and *Lpl*, responsible for the hydrolysis of triglyceride into fatty acid and glycerol, indicated that gymnemagenin upregulated the expression of *Lipe* and *Lpl* genes.

Adipogenesis is the process of differentiation of progenitor cells to mature adipocytes, and this structural change is essential to maintain physiological homeostasis. Dysregulation of adipogenesis leads to ectopic lipid deposition, lipodystrophy, systemic metabolic dysfunction, and an increased risk of developing diabetes and cardiovascular disease (Albert et al., 2014). Pparg is critical for adipogenesis, which controls the formation and size of lipid droplets in several ways, including the transcriptional regulation of *Plin2*, *Cidea*, and *Scd1*-lipid droplet scaffold protein genes. Several investigations have been reported on identifying therapeutic approaches targeting *Pparg* (Frkic et al., 2021). Phytochemicals have advantages over conventional drugs in that they are cost effective and may have fewer side effects than synthetic drugs. The present study reported that gymnemagenin upregulated the expression of *Pparg*, *Plin2*, *Cidea* and *Scd1*. The 100-ns molecular docking and dynamics studies identified that gymnemagenin strongly binds to the active site residues of the Pparg compared to its standard molecule, pioglitazone. This study can be further taken up for the identification of potential therapeutic targets and the use of gymnemagenin to treat obesity and T2DM.

Adipokines are the key modulators of lipid storage and distribution, appetite and satiety, and initiators of many immune responses (Kandasamy et al., 2012). In this study, it was found that gymnemagenin induces the upregulation of *Adipoq*, *Lep*, and *Ccl2*, which are very important adipokines in the regulation of lipid metabolism and insulin sensitivity. We also studied the gene expression pattern of *Fabp4* and *Slc2a4* upon gymnemagenin treatment. However, no significant change in gene expression was observed in either case. Similarly, from the molecular docking and dynamics studies, it is inferred that gymnemagenin has no affinity toward active site residues of Fabp4.

## 5 Conclusion

Gymnemagenin modulated six molecular pathways: AMPK, PPAR, insulin, Adipocytokine signalling and Fatty acid metabolism are considered to be two pathways, and regulation of lipolysis in adipocytes in *Homo sapiens*, *Mus musculus*, and *Rattus norvegicus*. An *in vitro* study showed that gymnemagenin escalated triglyceride metabolism by upregulating the expression of lipase genes *Lipe* and *Lpl,* responsible for the hydrolysis of triglycerides into fatty acid and glycerol. Gymnemagenin also upregulated the expression of the anti-inflammatory gene *Adipoq*. In addition, gymnemagenin treatment upregulated the expression of the *Pparg* gene and downstream target genes *Plin2*, *Cidea* and *Scd1*. Furthermore, this was supported by *in silico* data where molecular docking of gymnemagenin and standard drug pioglitazone with Pparg revealed that gymnemagenin scored lower binding energy than pioglitazone, and dynamics studies for 100 ns identified that gymnemagenin bound more strongly to the active site residues of the Pparg than its standard molecule pioglitazone. These findings indicated the importance of the therapeutic potency of gymnemagenin against the severe metabolic consequences of impaired structure and function of adipocytes and lipolysis leading to obesity and T2D.

## Data Availability

The datasets presented in this study can be found in online repositories. The names of the repository/repositories and accession number(s) can be found in the article/Supplementary Material.
