# Supplementary material for: Elucidating type 2 diabetes mellitus risk factor by promoting lipid metabolism with gymnemagenin: An in vitro and in silico approach
Source: Front Pharmacol. 2022 Dec 13;13:1074342. doi: 10.3389/fphar.2022.1074342 (PMC9792475; doi:10.3389/fphar.2022.1074342)
Supplement: Supplementary file 5 [file DataSheet1.DOCX]

Supplementary Figure


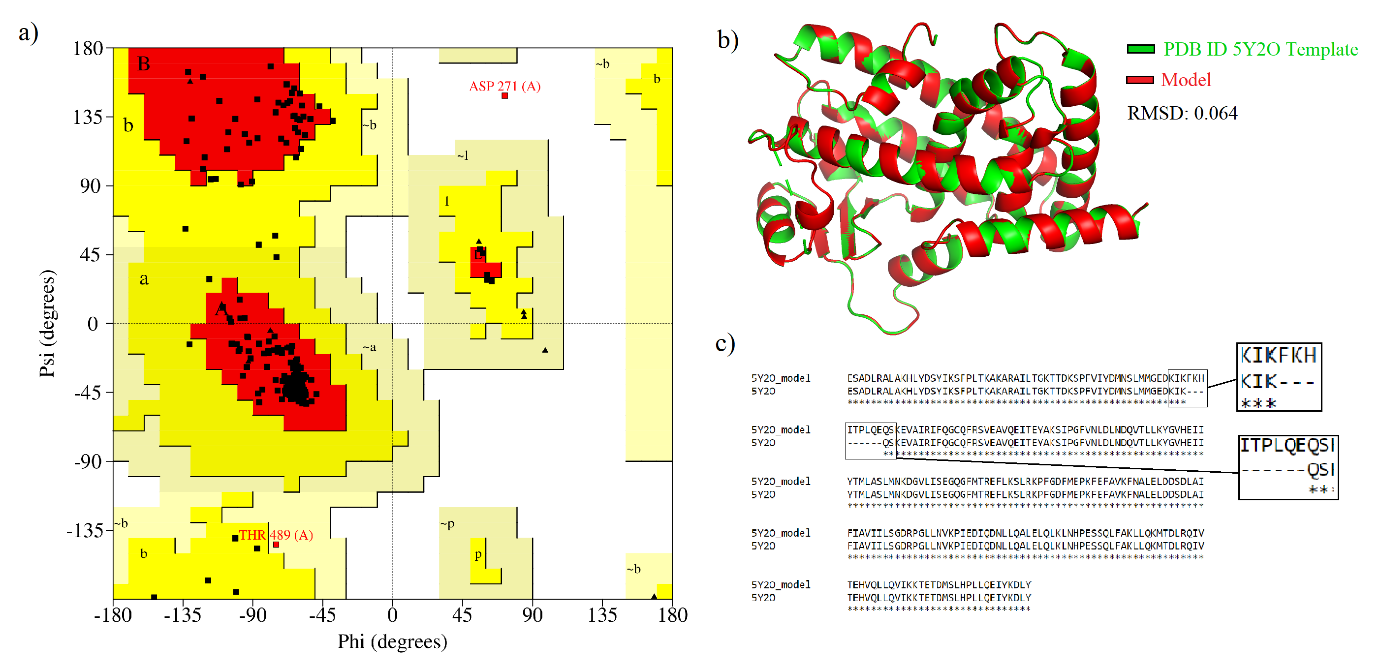


Supplementary Figure 1: Pparg model. a) Ramachandran plot for amino acid distribution, b) PyMOL superimpose (Green: Template (PDB ID: 5Y2O) and Red: Model), c) Clustal W template and model alignment.
